# Safety Assessment of the Extract of Phycobiliproteins Derived From *Arthrospira platensis*: Acute Toxicity Studies in Pacific Oysters

**DOI:** 10.1155/anu/2172814

**Published:** 2026-02-23

**Authors:** T. A. Kukhareva, A. A. Tkachuk, M. S. Podolskaya, A. B. Borovkov, E. S. Chelebieva, V. V. Parfenov, A. Yu. Andreyeva

**Affiliations:** ^1^ Laboratory of Ecological Immunology of Aquatic Organisms, A. O. Kovalevsky Institute of Biology of the Southern Seas of RAS, Moscow, 119991, Russia; ^2^ Department of Biotechnology and Phytoresources, A. O. Kovalevsky Institute of Biology of the Southern Seas of RAS, Moscow, 119991, Russia

**Keywords:** acute toxicity, DNA damage, heat shock proteins, hemocytes, mortality, phycobiliproteins, the Pacific oyster

## Abstract

Since shellfish farming has the potential to help feed a growing human population, it is crucial to anticipate new opportunities to improve the health of bivalves on farms and hatcheries. Phycobiliproteins (PBPs), natural nutritional components, are considered promising as immunomodulatory feed additives for aquaculture. The aim of this study was to examine the acute toxicity of a PBP extract obtained from *Arthrospira platensis* biomass for commercially important bivalve species, the Pacific oyster (*Magallana gigas* (Thunberg, 1793)). The PBP extract was added to water at final concentrations of 2, 20, and 200 μg/mL for a 24 and 48‐h exposure period. Compared to the control group, there were no treatment‐related biological effects on oyster mortality or induction of apoptosis or cellular death of hemocytes. However, exposure to the PBP extract significantly increased the respiratory rate of the oysters for 24–48 h. In the high‐dose group (200 μg/mL), a reduction in the activity of nonspecific cytoplasmic esterases and an increase in DNA damage levels in hemocytes were observed. Examinations of heat shock protein expression (HSP70 and HSP90) in the gills showed up‐regulation of HSP90 at a 200 μg/mL extract concentration after a 24 h exposure period and at all studied concentrations after 48 h of exposure. Although oysters in the high‐dose group displayed signs of genotoxicity and reduced nonspecific esterase activity of hemocytes, other parameters measured indicated low toxicity of the extract. The no‐observed‐adverse‐effect level of the PBP extract for adult Pacific oysters was determined as a concentration in water below 200 μg/mL.

## 1. Introduction

The northern and northeastern coast of the Black Sea has seen rapid development as a region for shellfish farming over the last few decades. The Pacific Oyster, *Magallana gigas* (Thunberg, 1793), is one of the most intensely farmed species of bivalve mollusks in global aquaculture, as well as in the Black Sea [1, 2]. However, the continuous development of marine aquaculture is challenged by several issues. One of these is general degradation of coastal aquatic systems due to climate change and human activity and the high density of shellfish farms in coastal areas, which can lead to reduced food supply. This can promote infections and parasitic invasions among bivalve mollusks on farms. Climate change has a significant impact on the spread of parasitism and diseases in freshwater and marine ecosystems [3, 4]. These disease and parasitic outbreaks can be extremely costly for businesses, as mass mortality may occur, reaching up to 100% of the population [5, 6]. Numerous studies have quantified the effects of environmental factors, including temperature, salinity, oxygen levels, and contaminants, on the immune system of bivalve mollusks [7]. The immune system of bivalves is relatively simple and is composed of innate nonspecific responses [8–10]. Hemolymph contains cells called hemocytes that are the main immune effectors, performing both cellular and humoral cytotoxic activities [9, 11–13]. Changes in environmental conditions can significantly reduce the cellular and humoral immunity of most commercial species. In response, new approaches to disease prevention have gained attention. One such approach is the use of bioactive immunomodulatory compounds in feed to improve the health of farmed marine organisms. This has the potential to contribute to sustainable aquaculture. The use of additives, such as probiotics, marine algae, and herbs, and their extracted compounds, which improve the resistance to diseases in aquatic animals by enhancing their innate defense mechanisms, is promising for the continued growth of the global aquaculture industry [14–16].

Modern regional and global oyster aquaculture requires the development of hatchery technologies to meet the needs of farms for seed material [17, 18]. The operation of oyster seed hatcheries should involve approaches that exhibit resistance to disease, rapid growth, and good health of spat and parents during broodstock conditioning. Oysters in hatcheries are subjected to a number of stressors imposed by aquaculture activities, including periodic desiccation in air and tumbling, which may reduce their health [19]. Water reservoirs and equipment on farms can also be potentially dangerous, as they may spread and transmit infections that affect oysters [20, 21]. Infectious disease outbreaks and reduced health status can have a negative impact on spawning efficacy [22]. During the period of broodstock conditioning, adult oysters are fed with a balanced diet, as the quality of feeding also affects their spawning [23, 24]. Therefore, the inclusion of biologically active compounds in the diet that have immunomodulating and antioxidant activity, and stimulate the nonspecific defense of the organism, is promising. Among the immunostimulating compounds that are considered possible candidates for sustainable aquaculture feeds, microalgae and their extracts have recently gained renewed attention [25]. Microalgae are known sources of various important biochemicals, including antioxidants, carotenoids, enzymes, lipids, minerals, etc. [26]. Due to their nutritional properties, several species of microalgae, as well as their extracts, are intensively studied and even applied in fish farming for health management [25]. For example, the implementation of metabolites from *Chlorella* and *Dunaliella* microalgae has improved the disease resistance and reduced mortality of shrimp post‐larvae [27]. Similarly, the spat of mussels (*Mytilus edulis* Linnaeus, 1758) and scallops (*Placopecten magellanicus* (Gmelin, 1791)) have demonstrated higher survival rates and improved physiological state when conditioned in water containing marennin, an extracellular pigment of diatomic algae *Haslea ostrearia* (Gaillon) Simonsen, 1974 [28]. The algal compounds significantly enhance the immune systems and disease resistance of commercially important fish species, such as rainbow trout (*Oncorhynchus mykiss* (Walbaum, 1792), gilthead seabream (*Sparus aurata* Linnaeus, 1758) and Senegalese sole (*Solea senegalensis* Kaup, 1858), among others, etc. [25].

Cyanobacterium *Arthrospira* (*Spirulina*) *platensis*, Gomont, 1892 is globally used as a source of various biologically active compounds, including phycobiliproteins (PBPs). One of the most commercially valuable PBPs is C‐phycocyanin, which can be used in food production, cosmetics, and pharmaceuticals [29–31]. PBPs have been intensively studied as a potential dietary supplement due to their potential health benefits. In vivo and in vitro studies on animal models have shown that these proteins possess a wide range of biological activities acting as antioxidants, immunomodulators, antitumor agents, and contributing to overall health improvement [32, 33]. Several toxicological reports have concluded that *A. platensis* is not toxic to animals [34–42]. However, there is limited information available on the toxicity of *Arthrospira* extracts, which are commercially distributed for use in aquaculture. Based on acute and subchronic toxicity studies conducted on spirulina extracts in rats [43, 44], no signs of toxicity were observed after up to 12 weeks of dietary administration. The potential adverse effects of commercially important aquatic invertebrates have not been thoroughly studied to date. In order to expand the toxicology database and increase confidence in the safety of *Arthrospira*’s extract for use in bivalve feed, the aim of this study was to assess potential acute toxic effects from administering an extract of *A. platensis* PBPs to Pacific oysters in a concentration range of 2–200 µg/mL in the water.

## 2. Materials and Methods

### 2.1. Animal Collection and Management

Adult Pacific oysters, *M. gigas*, (5 years old, weighing 72.1 ± 6.2 g, with a shell length of 10.8 ± 1.9 cm, *n* = 185), were collected in March 2024 from a shellfish farm (LLC “Maricultura” near Sevastopol (44.616014, 33.502248). The oysters were acclimatized to laboratory conditions for 1 week before the start of the experiments. The water in the 50–70 L tanks was maintained at 18–20°C, pH 8.2, oxygen concentration 7–8 mg/L, salinity 17–18 psu. During all stages of acclimation and experimentation, two‐thirds of the water volume was refreshed daily to ensure total metabolic waste removal. Oysters were fed daily with the microalgae species *Tetraselmis viridis* (Rouchijajnen) R.E.Norris, Hori & Chihara, 1980, strain IBSS‐25, from the collection of the Biotechnology and Phytoplankton Resources Department at the FRC IBSS. Feed was offered at equal proportions (109 cell microalgae species/day/animal). No oysters died during the acclimation period or during the experimental stages.

### 2.2. Extract Preparation

An aqueous extract of PBPs was obtained from the biomass of the cyanobacterium *A. platensis*. The culture of *A. platensis* was provided by the “Collection of aquatic organisms of the World Ocean” in FRC IBSS. Microalgae was grown on a Zarrouk medium in outdoor pools located in the greenhouses of the FRC IBSS experimental microalgae production facility.

The biomass was covered with a small amount of water and then frozen and thawed twice to destroy the cell membranes in order to facilitate rapid extraction of PBP. The pigments were extracted with cold distilled water (5°С) for 24 h. The extract was separated from the biomass by centrifugation (Eppendorf 5430R centrifuge, Germany, 7000 rpm for 10 min), and then stored in the dark at −18°C. Pigment concentrations were monitored spectrophotometrically (spectrophotometer UV‐2600i, Shimadzu Corporation, Japan) within wavelengths range 400–800 nm. Optical density was recorded at absorption maxima for R‐phycocyanin (615 nm), C‐phycocyanin (620 nm) and allophycocyanin (650 nm) and at 750 nm for estimating nonspecific absorption of the solution. Concentration of the pigments was estimated using the following equation [45] Equation (1):

(1) C-PBP =0.1660.091×D620−×D650,

where *D* is the optical density value for the corresponding wavelengths.

The concentration range of the extract was 4000–4500 μg/mL.

The yield of PBPs is 10 g of pigment from 100 g of biomass. The extract was purified by centrifugation at 7000 g. The D620/D280 ratio was used as an indicator of the purity of the extract. The D620/D280 ratio of 0.78 indicates that the extract is suitable for food use and is quite sufficient for use in aquaculture.

### 2.3. Experimental Design

**Figure 1:**
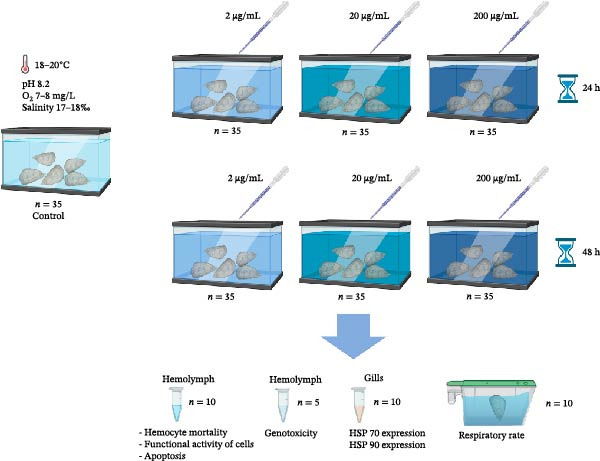
Principle schema of the experiment. Oysters were exposed to phycobiliproteins (final concentration in water 2, 20, and 200 μg/mL). And then toxicity markers were measured following 24 and 48 h of exposure.

For the acute toxicity test, oysters were randomly divided into four groups (three experimental and control) (*n* = 35) (Figure 1). During the experimentation, the oysters were not fed. Water solution of the extract was added to the tanks containing the oysters, and there were three different final concentrations of the extract in water: 2, 20, and 200 μg/mL. The range of concentrations used in this experiment was based on previous studies that did not find any adverse effects from the extract up to a concentration of 200 000 ppm [46]. The control group did not receive the extract. The oysters were kept in water with different concentrations of the extract for 24 and 48 h [47] After that, hemolymph samples were taken for flow cytometry (*n* = 10) and comet assays (*n* = 5), gill samples were collected for real‐time PCR analysis (*n* = 10) and frozen at **−**80°C, and the respiratory rate was measured (*n* = 10).

Hemolymph from the individual oyster (0.5–2.0 mL) was collected from the cardiac sinus using 1.0 mL sterile syringes. As hemocytes are adherent cells [48], hemolymph samples were kept on ice to prevent clumping. Plasma was removed by centrifugation using an Eppendorf 5430R centrifuge (Germany) at 500 g for 5 min at 10°C. After washing, the hemocytes were suspended in sterile sea water with a salinity of 17–18 psu and the cell concentration was adjusted to 2–4·10^7^ cells/mL.

### 2.4. Oxygen Consumption

Oxygen consumption by mollusks was determined using closed respirometry. Individuals were placed in hermetically sealed chambers, and the difference in oxygen concentration in the water was measured precisely over a defined period of 1 h. The full methodological details are described in our previous work [49]. Oysters (*n* = 10) from the experiment were carefully transferred to chambers. All measurements were taken 1 h after transferring the oysters into the respiration chamber and repeated three times (three replicates). Dissolved oxygen concentration was monitored for 1 h. Two respiration measurements were conducted in series on each group of oysters. Control measurements without mollusks were taken at the beginning and end of each respiration measurement series. Oxygen consumption was expressed as mg O_2_ g dry weight^−1^ h^−1^.

### 2.5. Flow Cytometric Measurements

The functional characteristics of hemocytes were assessed using flow cytometry. The measurements were conducted using a MACSQuant flow cytometer (Miltenyi Biotec, Germany). The data were processed using the manufacturer’s software (MACSQuantify Software, Germany). For each sample, 10,000 events were counted, and all measurements were repeated three times.

### 2.6. Hemocyte Mortality

The mortality of hemocytes was evaluated using propidium iodide (PI). The presence of this dye in the cells indicates a loss of membrane integrity, which is a sign of cell death [50, 51]) Cells were stained according to the protocol provided by the manufacturer. Briefly, 5 μL of a concentrated solution of PI (Sigma, St. Louis, MO, USA) was added to the 500 μL hemocyte suspension, resulting in a final concentration of 0.1 mg/L. The samples were then incubated in the dark for 30 min at 4°C. PI fluorescence excited at 488 nm wavelength was collected through a 575 nm band pass filter (FL3 channel) and data were registered on logarithmic scale. Cells presenting strong fluorescence represented dead cells. The number of dead cells was presented as a percent of total cell count in suspension.

### 2.7. Functional Activity of Cells

The functional activity of hemocytes was assessed based on nonspecific esterase activity using fluorescein diacetate, a nonspecific liposoluble substrate fluorescein diacetate (FDA, Molecular Probes, USA) [52]. 500 µL of a hemocyte suspension with FDA (final concentration of 0.1 mg/L in the probe) was incubated at room temperature in the dark for 15 min according to the recommendations provided by the manufacturer. Nonspecific esterase activity was expressed as mean fluorescence of FDA (FL1 channel) and expressed as percent of control fluorescence.

### 2.8. Apoptosis

To identify apoptosis in hemocytes (presence of phosphatidylserine on the outer leaflet of the plasma membrane), we used the Annexin V‐FITC apoptosis kit (BioVision, USA) as described by [53]. The hemocytes (500 µL) were processed with 5 µL Annexin‐V and after a 15 min incubation at 25°C in the dark. Cells stained positive with Annexin V‐FITC were considered in the end stage of apoptosis and the apoptosis‐associated fluorescence (FL1 channel) was measured.

### 2.9. RNA Extraction and qRT‐PCR: Quantification of Heat Shock Proteins (HSPs)

The total RNA from the gills of *M. gigas* was extracted using RNA Extran (Syntol, Russia) to assess the expression patterns of HSPs HSP70 and HSP90. After the exposure experiment was completed, 10 replicates of the gill samples were collected for each treatment group. The total RNA was purified and quantified in accordance with the manufacturer’s instructions. RNA samples were treated with DNase I mixture and purified RNAs were collected in 25 μL of nuclease‐free water (Evrogen, Russia). The quality and quantity of the RNA were analyzed using Qubit 4.0 (Thermo Fisher Scientific, USA), and the RNA samples were stained with ethidium bromide on a 1.5 % agarose gel. cDNA was synthesized using the Mint‐2 kit (Evrogen, Russia), following the manufacturer’s protocol. Reverse transcription was carried out in a final volume of 15 μL, containing 1 μg of purified RNA. All primer sequences used in this study are listed in Table 1 [54, 55].

**Table 1:** Real‐time quantitative PCR primers for heat shock proteins’ genes and elongation factor 1 α (EF1α) gene of the Pacific oyster *M. gigas*.

Gene name	Nucleotide sequence (5′‐3′)		Efficiency (%)	Genbank accession number
Elongation factor 1α (EF1α) (reference)	F	AGTCACCAAGGCTGCACAGAAAG	99.8	AB122066.1
	R	TCCGACGTATTTCTTTGCGATGT		
HSP70	F	AACGGTATCCTGAATGTGTC	101.1	AF144646
	R	CTTCTCGTCTTCCTGCTTG		
HSP90	F	CGAGGAAGCAGAAGCAGAG	98.8	EF687776.1
	R	ATGTCACCAGACGGTTAGATAC		

Quantitative real‐time PCR analysis was performed on a LightCycler 96 real‐time PCR instrument (Roche, Switzerland/Germany) using qPCRmix‐HS kit with SYBR Green I dye (Evrogen, Russia). Reaction mixture (total volume 25 µl) contained 1 µl of cDNA, 0.5 µM of each primer and Milli‐Q water. RT‐PCR was conducted as follows: initial denaturation at 95°C for 30 s followed by 40 cycles of denaturation at 95°C for 5 s, annealing at 55°C for 20 s, and extension at 72°C for 10 s and then 7 min at 72°C for final extension. Melting curve data were collected at 55–95°С (0.5°С/s). All reactions were performed with three replicates. For each run, a negative control without template was included. The data analysis was carried out using Roche software v.1.1 (Switzerland/Germany). Efficiencies of amplifications were determined by running a standard curve with serial dilutions of cDNA. For each measurement, a threshold cycle value (Ct) was determined as the fractional cycle number at which the fluorescence passes the fixed threshold. All data were expressed as relative to the elongation factor 1 α (EF1α) of *M. gigas*, which was used as a reference gene to normalize the expression levels between the samples after verification of stability during the experiment. The specificity of the real time RT‐PCR products was determined by melting curve analysis. Fold changes in the gene expression relative to the control were determined using the standard 2^−ΔΔCT^ method [56].

### 2.10. Comet Assay

The comet assay is a frequently employed technique for evaluating genotoxicity in environmental studies [57]. The analysis was conducted using the protocol developed by [58]. For each experimental group, samples of hemocytes were collected from five randomly chosen oysters, following the protocol outlined above. A suspension of hemocytes (40 µL) was mixed with 40 µL of 1% low‐melting agarose in phosphate‐buffered saline (PBS) and carefully layered on top of a layer of normal‐melting agarose (1% in PBS) on ice‐cold glass slides (ApexLab, Russia). A third layer was then created using 0.5% low‐melting agarose in PBS. The samples were incubated in lysing buffer at 4°C for 1 h in the dark. After that, the slides were transferred to an alkaline buffer (pH >12) and left there for 20 min. Electrophoresis was then performed under alkaline conditions (pH 10) for 20 min, followed by two rinses of the slides with ice‐cold distilled water. To visualize the comets, the cells were dyed with PI and observed under a fluorescent microscope (Olympus CX43, Japan). The analysis was conducted using CometScore software, version 1.5 (Tritek Technologies, Wilmington, Delaware, USA). The percentage of DNA in the tail was estimated for each sample as an indicator of DNA damage.

### 2.11. Statistics

Data are reported as means ± standard error (SE) unless other is stated. Statistical analyses were carried out using GraphPad Prism version 9 (GraphPad Software, USA). The data were first tested for normality using the Shapiro–Wilk’s test and for homogeneity of variance using Levene’s test. The mean comparison of quantitative variables compatible with a normal distribution was performed with two‐way ANOVA. Dunnett’s multiple comparisons test was used for comparing the differences between the experimental groups and the control group. Differences in the means were considered statistically significant when *p*  < 0.05.

Pearson’s correlation analysis was performed to explore the correlations between esterase activity in hemocytes and DNA damage levels in all groups. The heatmap was created in GraphPad Prism version 9 (GraphPad Software, USA), and *p*‐values less than 0.05 referred to significant differences ( ^∗^).

## 3. Results

### 3.1. Mortality

No mortality of oysters was observed in control and experimental groups throughout the entire period of the experiment.

### 3.2. Oxygen Consumption

Oxygen consumption was significantly affected by the addition of the extract, with an increase in oxygen consumption in oysters at all concentrations studied after 24 h of exposure (Figure 2). After 48 h of exposure, a significant effect on oxygen consumption was also observed. Oysters showed a dose‐dependent increase in oxygen consumption after experimental exposure to the extract. Oysters exposed to 200 μg/mL of the extract had more than three times higher levels of oxygen consumption compared to control groups (*p*  < 0.05).

Figure 2Oxygen consumption of *M. gigas* exposed to extract of phycobiliproteins derived from *A. plathensis* at (a) 24 h and (b) 48 h. Control – 0 μg/mL; Experimental groups: extract concentration in water 2, 20, and 200 μg/mL. Data presented as Mean ± SE (*n* = 10). Asterisks indicated significant differences ( ^∗∗^ *p*  < 0.01;  ^∗∗∗^ *p*  < 0.001) between extract‐treated and control groups at single time points.

**Figure:**
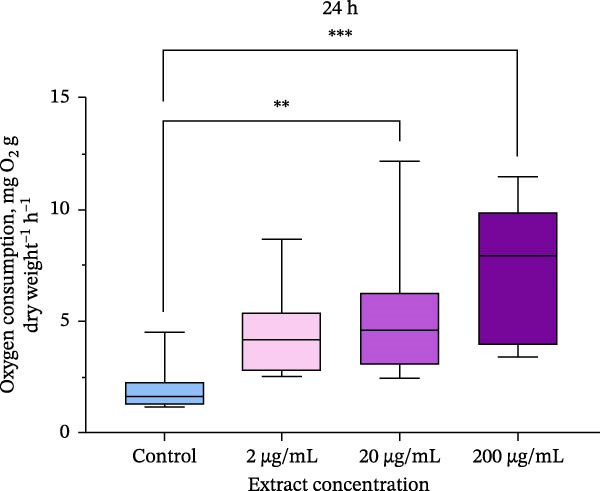
(a)

**Figure:**
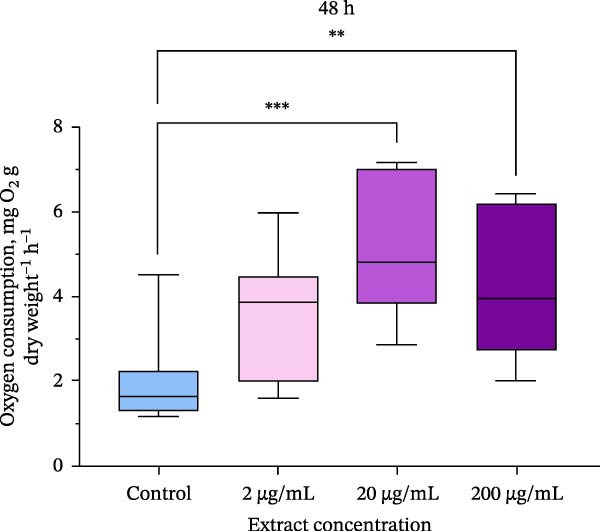
(b)

### 3.3. Hemocyte Activity and Mortality After Incubation of Oysters With PBP Extract

**Figure 3:**
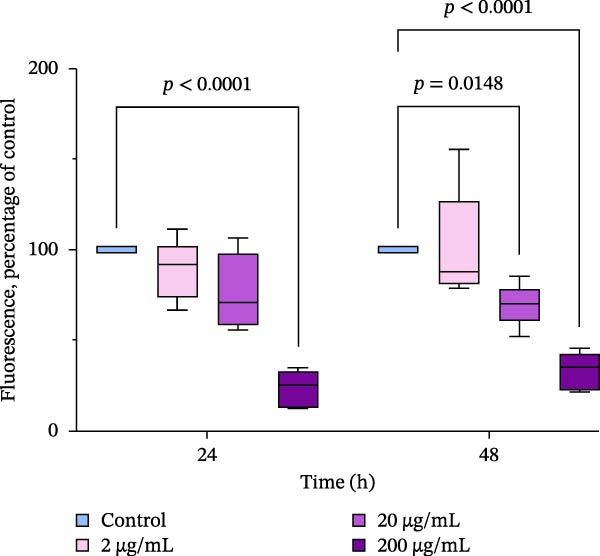
Nonspecific esterase activity of oyster hemocytes after the in vivo exposure to the PBP extract derived from *A. plathensis*. Control—0 μg/mL; Experimental groups: extract concentration in water 2, 20, 200 μg/mL. Data presented as Mean ± SE (*n* = 10) of folds of control FDA fluorescence at 24 and 48 h. Asterisks indicated significant differences (*p*  < 0.05) between extract‐treated and control groups at single time points.

The functional activity of hemocytes was assessed based on the activity of nonspecific esterases in the cytoplasm. Our data showed that the activity of esterases, as measured by fluorescence intensity (FDA), significantly decreased in oysters exposed to an extract at a concentration of 200 μg/mL for both 24 and 48 h of incubation. The FDA fluorescence levels were five times lower to those in the control group. In oysters exposed to the extract for 48 h, the first decrease in hemocyte viability was observed at a concentration of 20 μg/mL, and the second decrease peaked at 200 μg/mL of the extract in the water. (Figure 3)

After incubating oysters with PBP extract for 24–48 h, the number of dead cells in the hemolymph samples did not significantly change (Figure 4a). The average level of dead cells in the suspensions did not exceed 7.5 ± 1.3%.

Figure 4Relative levels of dead hemocytes (a) and DNA damage in hemocytes (b) after in vivo incubation of oysters with PBP extract derived from *A. plathensis*. Control—0 μg/mL; Experimental groups: extract concentration in water 2, 20, 200 μg/mL. The significant differences among the control and treated groups were subjected to two‐way ANOVA, followed by Dunnet multiple range test (*p* < 0.05).

**Figure:**
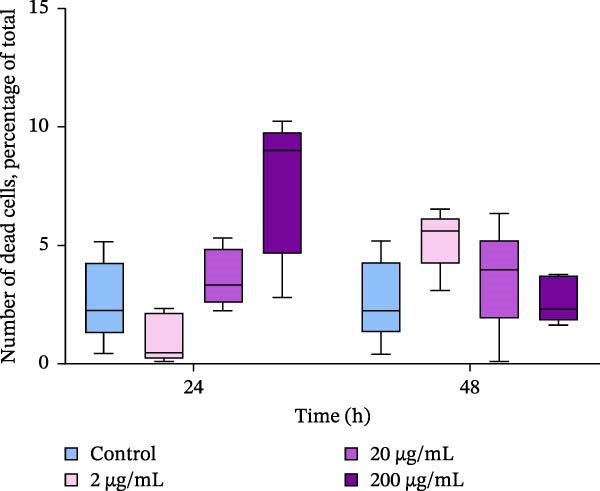
(a)

**Figure:**
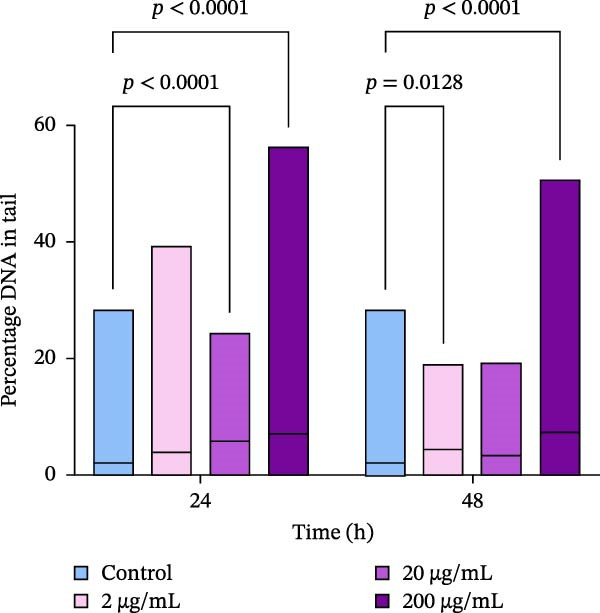
(b)

### 3.4. Apoptosis

**Figure 5:**
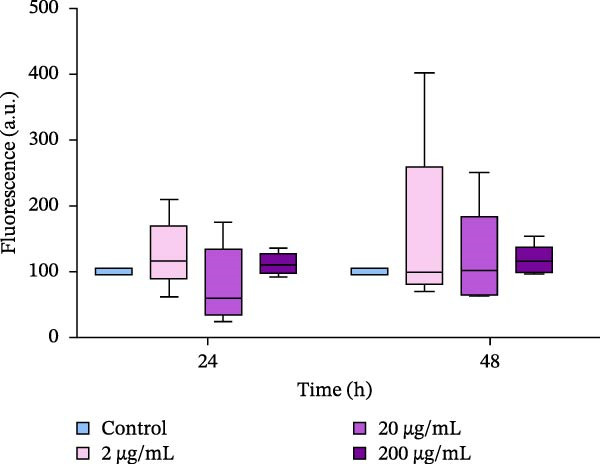
The number of apoptotic cells in suspensions of hemocytes after in vivo incubation of oysters with PBP extract derived from *A. plathensis*. Control—0 μg/mL; Experimental groups: extract concentration in water 2, 20, 200 μg/mL. The significant differences among the control and treated groups were subjected to two‐way ANOVA, followed by Dunnet multiple range test (*p* < 0.05).

Administration of the extract did not induce apoptosis in hemocyte cells throughout the entire period of the experiment. No significant differences between the control oysters and those exposed to the extract (2–200 μg/mL) were observed (Figure 5).

### 3.5. DNA Damage in Hemocytes

The genotoxicity of the extract was assessed using the comet assay method, and changes in DNA damage levels were defined as significant shifts in % DNA in the tail of the comet. A significant decrease in this parameter was not considered, as the control group was not exposed to any factors that could cause DNA damage in hemocytes. After 24 and 48 h of incubation of oysters with lower extract concentrations 2 and 20 µg/mL, no increase of DNA damage was observed in hemocytes. However, oysters incubated in water with 200 µg/mL of the extract showed significant increases in % DNA in the tail after 24 h (*p*  < 0.05; Figure 4b). The average increase in this parameter was 3.5‐fold. Similarly, there was an increase in DNA damage levels in hemocytes induced by a 200 μg/mL PBP extract after 48 h (*p*  < 0.05).

### 3.6. Expression of HSPs

Significant effects of the extract and time of exposure on the expression levels of HSP70 and HSP90 have been detected (*p*  < 0.05). Exposure of oysters to 2–20 µg/mL of the extract resulted in a decrease in the expression of HSP70 in the gills after 24 h of exposure (Figure 6a). However, after 48 h of incubation with the extract, the expression levels of HSP70 remained unchanged. In contrast, there was a strong increase in the expression of HSP90 within 24 h, with 200 µg/mL of the extract inducing an increase in HSP90 levels (Figure 6b). After 48 h of exposure, the expression levels of HSP90 were four to five times higher than the control levels (*p*  < 0.05).

Figure 6Relative mRNA expression levels of heat shock proteins’ genes in the gills of the Pacific oyster *M. gigas* fed with graded levels of extract of PBPs for 24 and 48 h: a—expression of HSP70; b—expression of HSP90 (two‐way ANOVA followed by Dunnet multiple range test).  ^∗^ Significantly different (*p*  < 0.05) compared with the control group.

**Figure:**
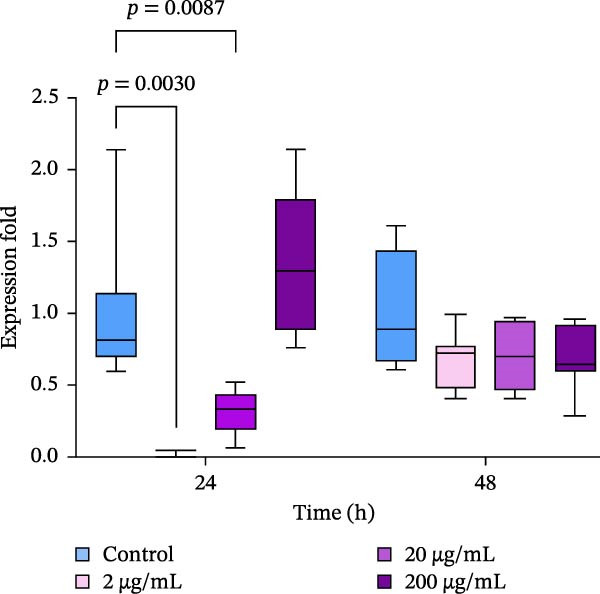
(a)

**Figure:**
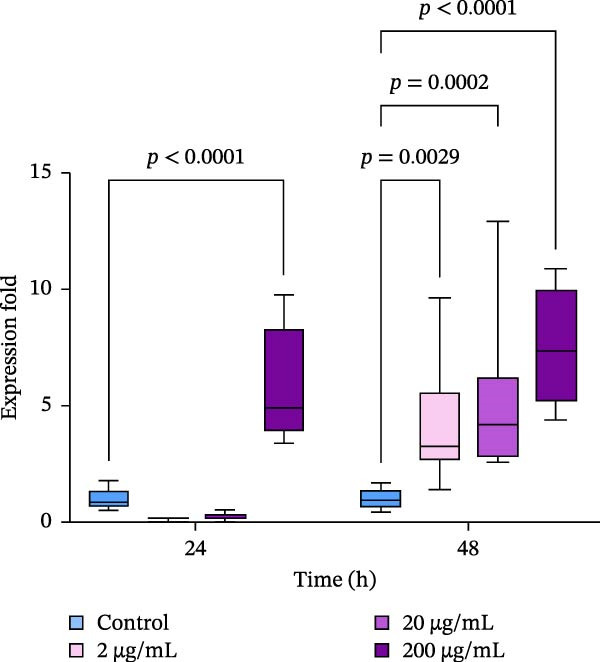
(b)

## 4. Discussion

The present study describes the results of several toxicity studies conducted to evaluate the safety of a novel extract of PBP derived from *A. plathensis* as a feed additive for oysters in aquaculture. The mortality test results did not show any significant changes due to the dissolving of the extract in water since no statistical differences between the treatments for any of the examined concentrations were observed.

In addition, oxygen consumption has been measured in oysters after exposure to different concentrations of the extract. Estimating oxygen consumption in bivalves is equivalent to quantifying metabolic expenditure and indirectly indicating filtration capacity of the organism [59]. An increase in oxygen consumption was observed at all extract concentrations during the entire experiment. Feed additives can affect oxygen consumption in marine organisms [60, 61]. Reports of elevated oxygen consumption with increased ration are common in many bivalve species, and have been attributed to acceleration of filtration rate and metabolism associated with increased feeding activity [62]. On the other hand, oxygen consumption in bivalves often increases under environmental contaminant exposure and may be used as a stress indicator [63, 64]. Based on the results of this study, observed increase in oyster oxygen consumption is interpreted as an indirect effect of enhanced feeding activity. This is inferred from the experimental conditions, which utilized the extract derived from spirulina that is recognized for its high nutritional quality [65]. We suppose that extract acted as a stimulant for filtration since the oxygen consumption increased gradually with the concentration of the extract added. The lack of mortality throughout the experiment suggests the oysters were in an active, nonstressed metabolic state, aligning with this hypothesis. It is important to note, however, that this study did not directly measure filtration rate, and thus, further research with targeted measurements over an extended period is required to substantiate this mechanism.

Where oysters are treated with the extract that has potential for use as an immune and antioxidant stimulant, it is important to understand its effects on the overall fitness of the organism. Measurement of hemocyte parameters can provide a good indication of physiological stress and health in bivalve mollusks [66, 67]. Hemocytes are the major immune‐effector cells, and therefore, changes in their function may affect the immune capacity of the organism [68, 69]. The proportion of dead hemocytes as well as presence of apoptotic hemocytes in the hemolymph of oysters exposed to the extract was similar to that of the control group. However, other parameters were affected by the presence of PBP extract in the water. Regarding hemocyte viability and the absence of signs of apoptosis induction, our experiments revealed a decrease in nonspecific esterase activity (measured using FDA dye) in oysters exposed to high concentrations of extract (200 µg/mL for a 24 h period and 20–200 µg/mL for 48 h). Cytoplasmic esterases play a role in the intracellular degradation of foreign particles [70, 71]. Therefore, a decrease in fluorescence observed in hemocytes after exposure of oysters to high concentrations of the extract may be due to a general disruption of cellular killing mechanisms and acute toxicity of high doses of the extract. This hypothesis is supported by elevated levels of DNA damage, which were accompanied by a decrease in hemocyte fluorescence in *M. gigas* after 24–48 h of exposure to 200 μg/mL extract. Such disturbances, including decreased hemocyte viability and increased markers of cellular damage (such as ROS production and apoptosis), have been observed in *M. gigas* hemocytes exposed to organic pollutants, including insecticides and polycyclic aromatic hydrocarbons (PAHs) [72–74]. The decrease in the activity of nonspecific esterases in hemocytes and the higher levels of DNA damage after 24–48 h (*p*  < 0.05) in oysters exposed to 200 μg/mL suggested toxic effects of the extract at high concentrations. However, the correlation analysis did not reveal any significant relation between DNA damage and esterase activity in hemocytes (Supporting Information 1: Figure S1), (Supporting Information 2: Figure S2). We also did not observe hemocyte mortality and apoptosis as well. However, based on our findings, we may hypothesize that the concentration of the PBPs extract 200 μg/mL could negatively affect oyster health after a short‐term exposure and corresponds to lowest observed adverse effect level (LOAEL).

The mRNA expression levels of HSPs genes in the gills of oysters were altered when they were exposed to different concentrations of the extract. According to the results of this study, the mRNA expression of HSP90 was significantly increased in all experimental groups, while the expression of HSP70 either remained unchanged or even decreased significantly compared to the control group. In aquaculture species, HSPs have been the subject of numerous studies because of their important role in reducing the stress‐induced denaturation of client proteins [75]. HSPs are also involved in protein folding, assembly, degradation, and regulation of gene expression [76, 77]. Physiological and environmental stressors such as high temperature, heavy metals, free radicals, air exposure, and microbial infection rapidly enhances the production of HSPs in immune cells and other tissues and organs of bivalves [22, 75, 78–80]. In particular, HSP70 was shown to play a crucial role in protecting cells against heat and other stresses [81]. In contrast to the results obtained in this study, bivalve mollusks exposed to various stressors, including toxins, usually show upregulation of HSP70 genes [82, 83]. However, downregulation of expression under stressful conditions is rarely observed. Therefore, it can be assumed that a short‐term (24 h) increase in HSP70 expression is unlikely to be caused by the administration of the extract and its toxicity. This assumption is supported by the lack of significant changes in other functional markers measured in hemocytes in these experimental groups, as well as by the stabilization of HSP70 expression during further exposure of the oysters to the extract (48 h) and its higher concentrations.

Recent studies have shown that HSPs (HSP90, HSP70, and HSP40) play a crucial role in mounting protective immune responses against bacterial and viral diseases [84, 85]. Thus, we may suppose that the upregulation of HSP90 upon short‐term administration of the extract could be a sign of immunomodulatory activity in gills since this tissue is one of the first barriers of the immune defense in bivalves [86]. This assumption is in line with the recent observation of up‐regulation of various HSP genes in aquatic invertebrates, including crustaceans and mollusks, in response to pathogen infection, challenge with gram‐negative or gram‐positive bacteria, etc. [78, 87–89] Moreover, the application of other biologically active compounds, such as plant‐based polyphenolic compounds like phloroglucinol and carvacrol, has also been shown to induce HSP70 expression and protect against bacterial infection in animals [90, 91]. Similarly, the application of dietary folic acid to blunt snout bream (*Megalobrama amblycephala* Yih, 1955) has also been shown to increase the expression of HSP60, HSP70, and HSP90, as well as enhance the acute high temperature resistance ability of the fish [92]. These findings, along with the results of this study, suggest that bioactive antioxidant and immunomodulatory compounds may highly trigger the expression levels of HSPs in the tissues of aquatic organisms. On the other hand, pronounced up‐regulation of HSP90 expression accompanied with decreased esterase activity in hemocytes and higher levels of DNA damage in hemocytes observed at 200 µg/mL of the extract is likely attributed to stress‐response of oysters.

In conclusion, the acute tolerance study revealed that the dietary extract of PBPs derived from *A. plathensis* shows low toxicity for adult Pacific oysters with “No Observed Adverse Effect Level (NOAEL)” in concentrations 2–20 µg/mL. The study did not reveal any significant changes in oyster mortality, induction of apoptosis or cellular death of hemocytes due to the dietary administration of the extract at concentrations ranging from 2 to 200 µg/mL. Acute toxicity assessed based on hemocyte functional parameters, including nonspecific esterase activity and the comet assay, showed a low level of safety concern for the extract in Pacific oysters at the concentrations of the extract between 2 and 20 µg/mL. However, reduced hemocyte viability and increased DNA damage in hemolymph cells were observed at a concentration of 200 µg/mL extract. The extract also modulated the expression of HSP90 mRNA in gills, indicating an immunomodulatory potential for the bioactive compound at moderate concentrations and stress‐response of oysters at concentrations 200 µg/mL. Additionally, it enhanced the oxygen consumption of oysters. Based on the results of this study, concentrations of the extract 200 μg/mL is suggested as LOAEL and is not recommended for further investigation of the potential immunomodulatory and antioxidant properties of the extract. To translate these laboratory results into an industrial application, future development should focus on functional feed formulations. The water‐soluble nature of PBP presents a challenge for incorporation into stable aquafeeds and for targeted delivery within the organism. Strategies such as microencapsulation or embedding the extract in lipid‐based carriers could be essential to protect these bioactive compounds from degradation during feed processing and storage, and to ensure their controlled release upon ingestion. Investigating such delivery systems will be a critical step in maximizing the efficacy and consistency of the extract as a commercial feed additive. However, further long‐term research is needed to investigate the immune status of adult Pacific oysters to confirm the immunomodulatory effects of the PBP extract.

## Author Contributions

**T.A. Kukhareva**: investigation, visualization, writing – original draft, conceptualization. **A.A. Tkachuk**: investigation, writing – original draft. **M.S. Podolskaya:** investigation, writing – original draft. **A.B. Borovkov**: investigation, writing – original draft, conceptualization. **E.S. Chelebieva:** investigation, writing – original draft, conceptualization. **V.V. Parfenov:** investigation, visualization, writing – original draft. **A. Yu. Andreyeva:** project administration, investigation, supervision, visualization, writing – review and editing, funding acquisition.

## Funding

This study was funded by the Russian Science Foundation, 24‐16‐00245.

## Conflicts of Interest

The authors declare no conflicts of interest.

## Supporting Information

Additional supporting information can be found online in the Supporting Information section.

## Supporting information


**Supporting Information 1** Figure S1. Correlation matrix of DNA damage and esterase activity in hemocytes of *Magallana gigas* exposed to aqueous phycobiliprotein extract for 24 h. The heatmap depicts the Pearson correlation coefficients (*r*) between the level of DNA damage (measured as % DNA in tail using the Comet assay) and esterase activity (measured as mean fluorescence intensity of hydrolyzed FDA) in hemocytes. Each cell contains the numerical correlation coefficient (*r*‐value). The asterisks denote the statistical significance of the correlation ( ^∗^ *p* < 0.05).

**Supporting Information 2** Figure S2. Correlation matrix of DNA damage and esterase activity in hemocytes of *Magallana gigas* exposed to aqueous phycobiliprotein extract for 48 h. The heatmap depicts the Pearson correlation coefficients (*r*) between the level of DNA damage (measured as % DNA in tail using the Comet assay) and esterase activity (measured as mean fluorescence intensity of hydrolyzed FDA) in hemocytes. Each cell contains the numerical correlation coefficient (*r*‐value). The asterisks denote the statistical significance of the correlation ( ^∗^ *p* < 0.05).

## Data Availability

The data that support the findings of this study are available from the corresponding author upon reasonable request.
