# Supplementary figures and images for: Safety Assessment of the Extract of Phycobiliproteins Derived From Arthrospira platensis: Acute Toxicity Studies in Pacific Oysters
Source: Aquac Nutr. 2026 Feb 23;2026:2172814. doi: 10.1155/anu/2172814 (PMC12927954; doi:10.1155/anu/2172814)

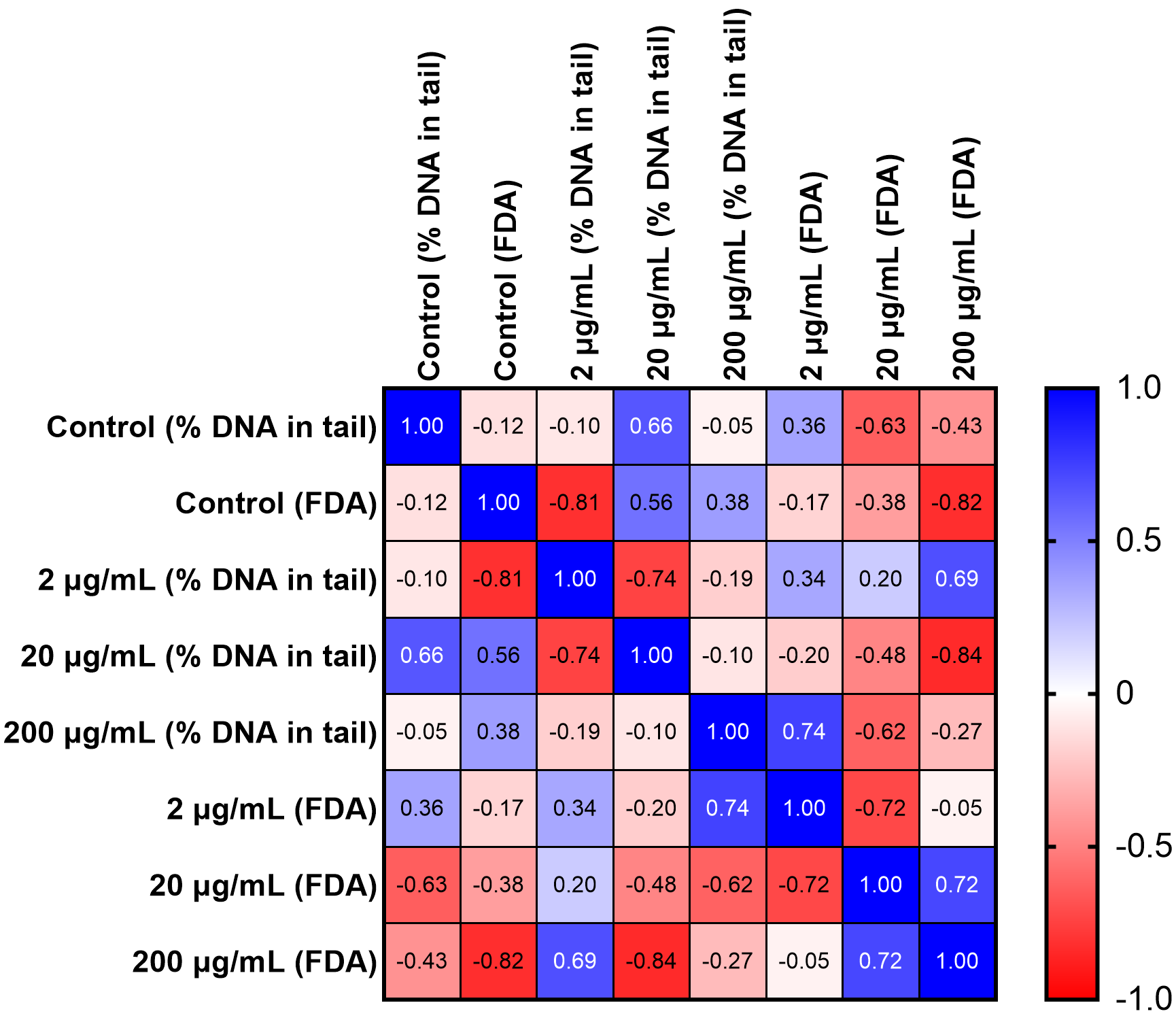

Supplement: Supplementary file 1 — Supporting Information 1 Figure S1. Correlation matrix of DNA damage and esterase activity in hemocytes of Magallana gigas exposed to aqueous phycobiliprotein extract for 24 h. The heatmap depicts the Pearson correlation coefficients (r) between the level of DNA damage (measured as % DNA in tail using the Comet assay) and esterase activity (measured as mean fluorescence intensity of hydrolyzed FDA) in hemocytes. Each cell contains the numerical correlation coefficient (r‐value). The asterisks denote the statistical significance of the correlation ( ∗ p < 0.05). [file ANU-2026-2172814-s001.tif]

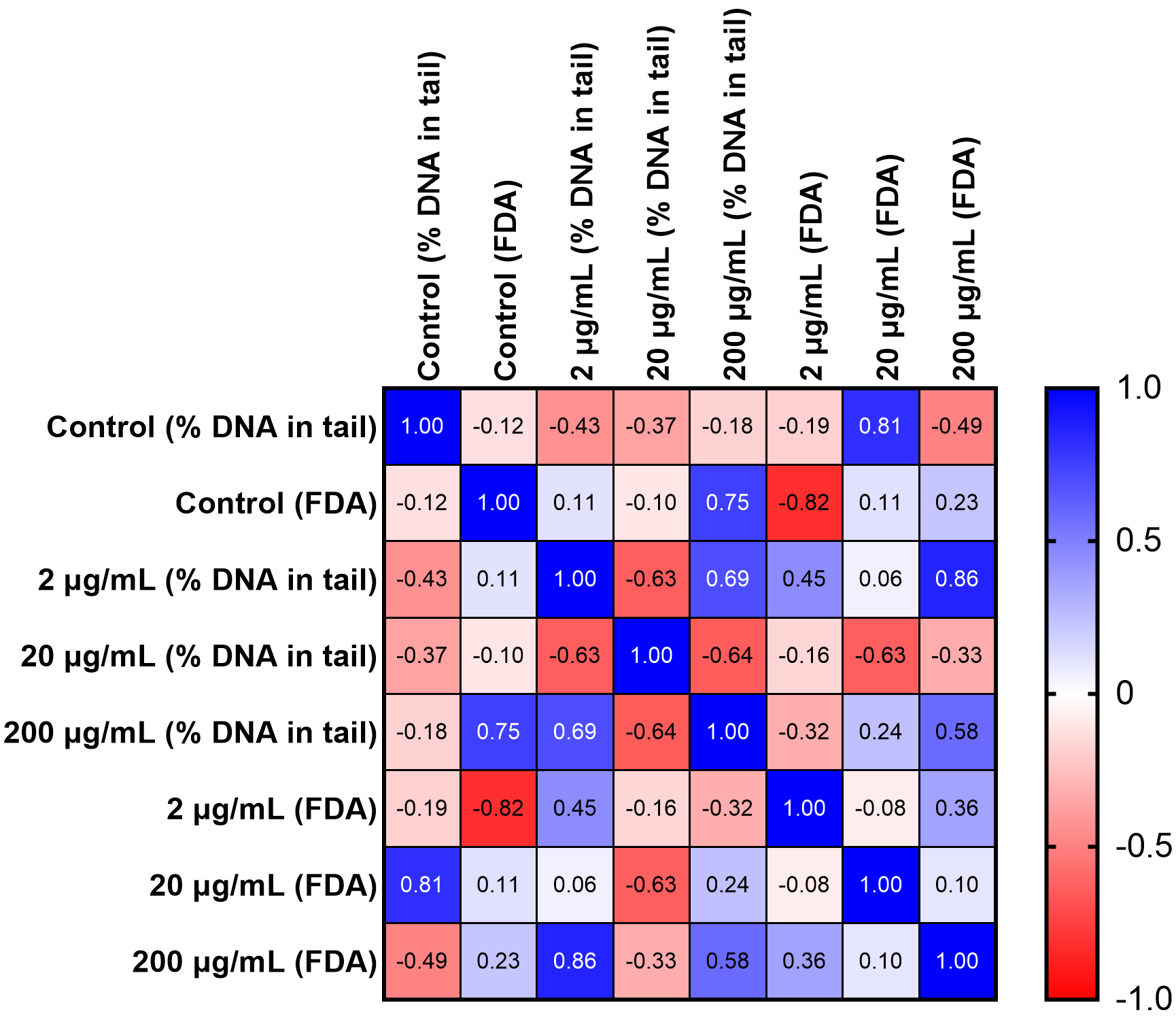

Supplement: Supplementary file 2 — Supporting Information 2 Figure S2. Correlation matrix of DNA damage and esterase activity in hemocytes of Magallana gigas exposed to aqueous phycobiliprotein extract for 48 h. The heatmap depicts the Pearson correlation coefficients (r) between the level of DNA damage (measured as % DNA in tail using the Comet assay) and esterase activity (measured as mean fluorescence intensity of hydrolyzed FDA) in hemocytes. Each cell contains the numerical correlation coefficient (r‐value). The asterisks denote the statistical significance of the correlation ( ∗ p < 0.05). [file ANU-2026-2172814-s002.tif]
